# Supplementary material for: Experiencing Food Restrictions for Health and Weight Control in Childhood and Their Links to Restrained Eating and Excessive Body Weight in Polish Young Adults—A Cross-Sectional Study
Source: Nutrients. 2024 Dec 29;17(1):87. doi: 10.3390/nu17010087 (PMC11723111; doi:10.3390/nu17010087)
Supplement: Supplementary file 1 [file nutrients-17-00087-s001.zip › nutrients-3371851-supplementary.pdf]

Questionnaire

1. To what extent do you agree or disagree with the sentences describing family habits from the period of your childhood (when you were 5-10 years old)

   Please choose for each statement one of the following answers:

   1 – disagree
   2 – slightly disagree
   3 – neither agree nor disagree
   4 – slightly agree
   5 – agree
   6 – I don't remember

___ My parents took care of me not eating too many high-fat foods (restriction for weight control).
___ My parents encouraged me to eat less so I won't get fat (restriction for weight control).
___ My parents gave me small helpings of food to control my body weight (restriction for weight control).
___ If I ate more at one meal, my parents tried to decrease my food helpings at the next meal (restriction for weight control).
___ My parents restricted the foods that would possibly make me gain weight (restriction for weight control).
___ My parents believed that there are certain foods that I should not consume to prevent weight gain (restriction for weight control) .
___ My parents did not allow me to eat between meals because they didn't want me to gain weight (restriction for weight control).
___ My parents have often put me on a diet to control my weight (restriction for weight control).
___ If my parents did not control my eating, I would have eaten more "unhealthy foods" (restriction for health).
___ If my parents did not control my eating, I would have eaten more of my favorite foods (restriction for health).
___ My parents wanted to ensure I did not overeat my favorite foods (restriction for health).
___ My parents wanted to ensure I did not eat too many sweets (restriction for health).

2. Using the scale below, please indicate the number that best describes your answer to each question:

   1 - Never
   2 - Seldom
   3 - Sometimes
   4 - Often
   5 - Very often

___ If you have put on weight, do you eat less than you usually do?
___ Do you try to eat less at mealtimes than you would like to eat?
___ How often do you refuse food or drink offered because you are concerned about your weight?
___ Do you watch exactly what you eat?
___ Do you deliberately eat foods that are slimming?
___ When you have eaten too much, do you eat less than usual the following days?'
___ Do you deliberately eat less in order not to become heavier?
___ How often do you try not to eat between meals because you are watching your weight?
___ How often in the evening do you try not to eat because you are watching your weight?
___ Do you take into account your weight with what you eat?

3. What restrictions do you apply to your food intake? Please choose for each statement one of the following answers:
   1 – Yes
   2 - No

___ I restrict highly processed products
___ I restrict sweets
___ I restrict high-fat products
___ I restrict high-sugar products
___ I restrict products containing preservatives, emulsifiers, etc.
___ I restrict other (which?) ……………
___ I do not apply any restrictions.
